# Insomnia Symptoms Are Associated with Measures of Functional Deterioration and Dementia Status in Adults with Down Syndrome at High Risk for Alzheimer’s Disease

**DOI:** 10.3233/JAD-220750

**Published:** 2024-07-16

**Authors:** Shivum Desai, Ivy Y. Chen, Christy Hom, Eric Doran, Dana D. Nguyen, Ruth M. Benca, Ira T. Lott, Bryce A. Mander

**Affiliations:** a Department of Pediatrics, University of California, Irvine, CA, USA; b Ascension Providence Hospital, Michigan State University College of Human Medicine, Southfield, MI, USA; c Department of Psychiatry and Human Behavior, University of California, Irvine, CA, USA; d Center for the Neurobiology of Learning and Memory, University of California, Irvine, CA, USA; e Institute for Memory Impairments and Neurological Disorders, University of California, Irvine, CA, USA; f Department of Cognitive Sciences, University of California, Irvine, CA, USA; g Department of Psychiatry and Behavioral Medicine, Wake Forest University, Winston-Salem, NC, USA

**Keywords:** Activities of daily living, Alzheimer’s disease, dementia, disorders of excessive somnolence, Down syndrome, sleep, sleep apnea syndromes, sleep initiation and maintenance disorders

## Abstract

**Background:**

While obstructive sleep apnea (OSA) and insomnia symptoms in neurotypical populations are associated with Alzheimer’s disease (AD), their association with dementia in adults with Down syndrome (DS) remains less clear, even though these symptoms are prevalent and treatable in DS. Understanding their associations with AD-related dementia status, cognitive impairment, and functional deterioration may lead to interventions to slow decline or disease progression in adults with DS.

**Objective:**

To characterize differences in OSA and insomnia symptom expression by dementia status, and to determine which sleep factors support dementia diagnosis.

**Methods:**

Multimodal consensus conference was used to determine dementia status in 52 adults with DS (52.2 ± 6.4 years, 21 women). Cognitive impairment, adaptive behavior skills, and symptoms of OSA and insomnia were quantified using validated assessments for adults with DS and their primary informants.

**Results:**

A sex by dementia status interaction demonstrated that older women with DS and dementia had more severe terminal insomnia but not OSA symptoms relative to older women with DS who were cognitively stable (CS). Greater insomnia symptom severity was associated with greater functional impairments in social and self-care domains adjusting for age, sex, premorbid intellectual impairment, and dementia status.

**Conclusions:**

Insomnia symptoms are more severe in women with DS with dementia than in women with DS and no dementia, and regardless of dementia status or sex, more severe insomnia symptoms are associated with greater impairment in activities of daily living. These findings underscore the potential importance of early insomnia symptom evaluation and treatment in women with DS at risk of developing AD.

## INTRODUCTION

Down syndrome (DS) is a genetic disorder caused by the triplication of all or part of chromosome 21 [1–3], which contributes to overexpression of the amyloid-β protein precursor (AβPP) gene. This results in increased amyloid-β pathological accumulation that leads to an early and ubiquitous onset of Alzheimer’s disease (AD) neuropathology relative to neurotypical populations [4–6].

In neurotypical older adults, multiple forms of dementia including AD are associated with sleep disturbance, with symptoms of sleep-disordered breathing, excessive daytime sleepiness, and insomnia each being observed in nearly half of patients [7]. Findings from a recent meta-analysis indicate that patients with AD are five times more likely to have obstructive sleep apnea (OSA) than healthy older adult controls [8]. The high prevalence of sleep disturbance in patients with AD is clinically relevant. These various forms of sleep disturbance are associated with worsening cognitive and behavioral functioning, emergence of psychiatric symptoms, and poor quality of life [9–11]. Moreover, the presence of nighttime sleep disturbances is among the most frequent reasons given for institutionalization of patients with AD [12].

In the neurotypical population, even prior to the onset of AD, sleep disturbances have been shown to be associated with AD pathophysiology and increased risk for developing dementia [6–16]. Specifically, OSA, insomnia symptoms, and presence of excessive daytime sleepiness are all associated with higher cortical amyloid-β burden [13–15], while OSA treatment improves cognition in patients with AD and reduces AD biomarkers in cerebrospinal fluid [9, 16].

Although sleep disorders such as OSA and insomnia disorder are common in individuals with DS from an early age [17–20], they are often underdiagnosed in this population [21]. More than 65% of adults with DS are estimated to have OSA [21], excessive daytime sleepiness and insomnia are also prevalent, with rates over 70% being reported in some studies [19, 21, 22]. However, there is little information on the possible association between sleep disturbances and dementia as manifested by cognitive and functional decline. This study was designed to examine these relationships.

We tested the hypothesis that OSA and insomnia symptoms are more severe in adults with DS who have probable AD dementia compared to those who are cognitively stable (CS). We further studied relationships between OSA and insomnia symptoms with manifestations of dementia, including impairments in cognitive functioning and activities of daily living (ADLs).

## MATERIALS AND METHODS

### Study protocol

Sleep disorder symptoms (i.e., sleep apnea risk, excessive daytime sleepiness, and insomnia symptom severity) were assessed from standardized caregiver reports for adults with DS enrolled at one site of the Alzheimer Biomarkers Consortium— Down Syndrome (ABC-DS) study, a multi-site, longitudinal AD biomarker study (AG051420) [23]. Demographic data, consensus diagnosis of dementia status, and outcomes on dementia-related cognitive and adaptive behavior instruments were obtained from the ABC-DS study [24–26]. Due to all of our participants having lifelong intellectual impairment [27], data were collected through a combination of direct testing and structured interviews with participants’ caregivers [28–30]. The sleep questionnaires were collected independently from the consensus diagnostic data and a review of the AD biomarker data, during two time points of the longitudinal study (i.e., during cycles 1 and 2). These measures were recorded prior to the Coronavirus Disease 2019 (COVID-19) pandemic. Most sleep data (>92%) were collected during cycle 1 data collection, and all sleep data were paired with data from their respective cycle for analyses. Twenty-nine of the informants were family members and 23 were paid staff caregivers. All but 3 informants were the participants’ primary caretaker. This study was in compliance with the Declaration of Helsinki and was approved by the Institutional Review Board of the University of California, Irvine. Informed assent and consent were obtained from all participants and their caregivers.

### Cohort

Demographic and clinical characteristics of the study population (*n* = 52) are shown in Tables 1 and 2, which was largely representative of the ABC-DS cohort (*N* = 304). Demographic and clinical characteristics of the current cohort compared to the ABC-DS sample at cycle 1 and cycle 2 are presented in Supplementary Tables 1 and 2, respectively. The current cohort was older and fewer participants had a community paid job. There were no significant cohort differences in proportions of participants with dementia, and there were no significant differences in breakdowns by sex, premorbid intellectual impairment, form of residence, or participation in a day program. Eligibility criteria for the current study included age ≥40 years, a clinical diagnosis of DS [31, 32], stable medical status and stable use of medications including psychotropic agents for at least 3 months. Pregnant participants and those with advanced dementia were excluded. Premorbid intellectual impairment (PII) was obtained from a review of past medical records by trained staff, and classified using ICD-10 criteria for mild, moderate, severe, or profound intellectual disability (ID) [33]. Chart-based review of intellectual disability level included data from the Wechsler Intelligence Scale for Children [34, 35], Wechsler Adult Intelligence Scale [36], or Stanford-Binet [37] intelligent quotient scores prior to dementia onset. Due to the limited number of study participants with severe PII (*n* = 3), analyses in the current study stratified PII as mild versus moderate and severe.

**Table 1:** Cohort demographic characteristics

	All participants (*n* = 52)	CS (*n* = 40)	DSAD (*n* = 12)		
	Mean±SD	Mean±SD	Mean±SD	*t*, *χ*^2^	*p*
Age (years)	52.2 ± 6.4	51.2 ± 6.1	55.5 ± 6.5	–2.09	0.042
Sex				4.48	0.034
Male (*n*; %)	31 (59.6)	27 (67.5)	4 (33.3)		
Female (*n*; %)	21 (40.4)	13 (32.5)	8 (66.7)		
Body Mass Index (kg/m^2^)	30.0 ± 5.9	30.0 ± 5.3	30.9 ± 8.0	–0.36	0.725
PII				0.66	0.417
Mild (*n*; %)	25 (48.1)	18 (45.0)	7 (58.3)		
Moderate (*n*; %)	24 (46.2)	21 (52.5)	3 (25.0)		
Severe (*n*; %)	3 (5.8)	1 (2.5)	2 (16.7)		
Residence				3.91	0.142
With family/caregiver (*n*; %)	26 (50)	23 (57.5)	3 (25.0)		
Agency/group home (*n*; %)	20 (38.5)	13 (32.5)	7 (58.3)		
Independent (*n*; %)	6 (11.5)	4 (10.0)	2 (16.7)		
Day program				1.53	0.216
Yes (*n*; %)	31 (59.6)	22 (55.0)	9 (75.0)		
No (*n*; %)	21 (40.4)	18 (45.0)	3 (25.0)		
Community paid job				0.07	0.797
Yes (*n*; %)	10 (19.2)	8 (20.0)	2 (16.7)		
No (*n*; %)	42 (80.8)	32 (80.0)	10 (83.3)		
Sleep disorder diagnosis				0.42	0.812
None (*n*; %)	40 (76.9)	31 (77.5)	9 (75.0)		
Sleep apnea (*n*; %)	11 (21.2)	8 (20.0)	3 (25.0)		
Insomnia (*n*; %)	1 (1.9)	1 (2.5)	0 (0)		

DSAD, Down syndrome-associated Alzheimer’s disease; CS, cognitively stable; PII, premorbid intellectual impairment.

**Table 2:** Cohort clinical characteristics

	All participants (*n* = 52)	CS (*n* = 40)	DSAD (*n* = 12)		
	Mean±SD	Mean±SD	Mean±SD	*t*, c^2^	*p*
DSMSE	63.0 ± 20.8	68.1 ± 18.9	44.5 ± 17.4	3.73	0.001
DLD SCS	10.8 ± 11.4	7.0 ± 8.3	23.7 ± 10.9	–5.68	<0.001
DLD SOS	9.3 ± 7.9	7.0 ± 5.5	17.0 ± 9.7	–3.42	0.004
Stress Index					
Total impact of events	3.88 ± 3.13	3.88 ± 3.19	3.92 ± 3.06	–0.040	0.968
Total number of events	1.85 ± 1.41	1.83 ± 1.41	1.92 ± 1.44	–0.196	0.845
VABS-3 scores					
Adaptive behavior composite	35.0 ± 17.2	37.7 ± 17.9	26.3 ± 11.5	2.59	0.015
C: Receptive language	62.0 ± 16.1	66.7 ± 11.2	46.3 ± 20.2	3.35	0.005
C: Expressive language	75.5 ± 24.3	79.3 ± 21.6	63.1 ± 29.3	2.09	0.041
C: Written language	31.5 ± 17.2	33.3 ± 15.8	25.5 ± 20.7	1.40	0.169
DLS: Personal	87.2 ± 20.2	93.3 ± 13.2	66.9 ± 26.3	3.35	0.005
DLS: Domestic	32.8 ± 17.2	36.2 ± 16.3	21.3 ± 15.3	2.82	0.007
DLS: Community	49.7 ± 29.9	54.1 ± 29.3	35.0 ± 28.0	1.99	0.052
S: Interpersonal relationships	63.3 ± 21.1	66.4 ± 19.2	53.1 ± 24.8	1.97	0.055
S: Play & leisure	41.9 ± 20.8	44.0 ± 19.7	35.0 ± 23.9	1.32	0.192
S: Coping	44.3 ± 15.8	48.2 ± 13.3	31.4 ± 17.0	3.58	0.001

DSAD, Down Syndrome-associated Alzheimer’s disease; CS, cognitively stable; DSMSE, Down Syndrome Mental Status Exam; DLD, Dementia Questionnaire for People With Learning Disabilities; SCS, Sum of Cognitive Scores; SOS, Sum of Social Scores; VABS-3, Vineland Adaptive Behavior Scales, 3^rd^ Edition; C, Communication; DLS, Daily Living Skills; S, Socialization. DSMSE and VABS-3 scores represent ability with higher scores indicating greater functioning. DLD scores represent impairment with higher scores indicating lower functioning.

### Sleep-related assessments

Past diagnoses of sleep disorders, sleep disorder treatment, and sleep disorder treatment adherence were collected through caregiver interviews and a review of past medical records (Table 1). Past diagnosis of OSA was adjusted for in analyses to avoid bias on reported outcomes and their associations with treatment and OSA symptom reporting.

The STOP-Bang questionnaire is a validated 8-item questionnaire assessing an individual’s risk of having OSA [38, 39], and has been used in adults with DS and validated against polysomnography [40]. Questions about snoring, tiredness, observed apnea, high blood pressure, body mass index >35 kg/m^2^, age >50 years, neck circumference >16 inches, and male sex are scored as Yes/No answers, and composite scores range from 0 to 8. A score of 3 or more indicates intermediate to high risk of having OSA [38].

The Epworth Sleepiness Scale (ESS) quantifies participants’ level of excessive daytime sleepiness [41], which is often associated with symptomatic OSA [41, 42]. This instrument rates the probability of falling asleep in various situations encountered in daily life on a 4-point Likert Scale (0 = *would never doze* to 3 = *high chance of dozing*). Due to the cognitive impairments of individuals with DS, two items that were not applicable to this population were replaced: “Sitting and reading” was modified to “Sitting and looking at a picture book or electronic device (e.g., iPad, iPhone, tablet, Kindle, or handheld game device)” because reading ability is limited in DS. The item “In a car, while stopped for a few minutes in traffic” was changed to “Sitting and eating a meal”. This latter item was taken from the ESS for Children and Adolescents (ESS-CHAD) [43], since many individuals with DS do not drive and an assessment that was comparable across all participants was necessary. This new item replicates a similar scenario of the original item that requires active attention. A composite score was created ranging from 0 to 24, with scores > 10 indicating the presence of excessive daytime sleepiness [41].

The Child Sleep Habits Questionnaire-Abbreviated (CSHQ-A) is an abbreviated survey used by the National Institute of Child Health and Development’s Study of Early Child Care and Youth Development designed for parental informants of school-aged children [44]. The CSHQ-A has been used as a sleep screening instrument to identify both behaviorally- and medically-based sleep problems in school-aged children, including symptoms of insomnia [45]. This questionnaire has been validated for use in individuals with DS [46], and items focused on insomnia symptoms. Based on the Diagnostic and Statistical Manual of Mental Disorders, Fifth Edition (DSM-5) criteria of insomnia disorder [47], three items were extracted from the CSHQ-A and utilized in the current study to assess observed insomnia symptomatology characterized by problems with sleep onset (difficulty falling asleep within 30 min), sleep maintenance (waking up frequently during the night and having difficulty falling back asleep), or early morning awakenings (terminal insomnia; waking up earlier than necessary or desired in the morning and having difficulty falling back asleep). Each item (sleep onset insomnia, sleep maintenance, terminal insomnia) was rated on a 5-point scale according to the weekly frequency of symptoms over the past three months [0 = *never (0 days/week)* to 4 = *always (7 days/week)* ]. A composite insomnia symptom severity total score was derived by summing the scores from each item (i.e., score range 0–12).

A Stress Index questionnaire [23] was used to account for potential confounding effects of stressful life events on sleep and behavioral measures. The number of events/changes within the last 12 months related to living arrangement, employment, and participant and family health status were used to form two variables: 1) the total number of stressful events that were recorded and 2) the impact of the stressful events on the participant (EVENTS IMPACT; handled well, handled badly, no impact, complex impact).

### Cognitive and adaptive behavior assessments

The Down Syndrome Mental Status Exam (DSMSE) is a direct assessment of cognitive function in adults with DS in multiple domains, including orientation, short-term memory, autobiographical memory, language, visuospatial function, and praxis [25]. A total score (ranging from 0–76) is computed, with lower scores indicating greater cognitive impairment.

The Dementia Questionnaire for People with Learning Disabilities (DLD) is an informant-based screening tool that was developed for early detection of dementia in adults with pre-existing cognitive impairments [24]. The DLD consists of eight subscales: short-term memory, long-term memory, orientation, speech, practical skills, mood, activity and interest, and behavioral disturbance. A total of 50 items are scored from 0 to 2 (0 = no problem, 1 = moderate problem, 2 = severe problem) to create two composite scores. The Sum of Cognitive Score (SCS) is comprised of the short-term memory, long-term memory, and orientation subscales, and is an indirect assessment of cognitive impairment. The Sum of Social Score (SOS) is comprised of the speech, practical skills, mood, activity and interest, and behavioral disturbance subscales, and is an indirect assessment of social impairment. Higher scores indicate greater impairment.

The Vineland Adaptive Behavior Scales, 3^rd^ edition (VABS-3) is a semi-structured, informant-based interview that measures adaptive behavior function [26]. This scale is sensitive to neurodevelopmental abnormalities and is often used to aid clinical diagnosis [48–50]. It measures an individual’s ability to perform everyday behaviors independently in comparison to other individuals of the same age in three domains: communication, daily living skills, and socialization. Items were scored on a scale of 0 to 2 (0: the participant does not perform the described behavior independently, 1: the participant only performs the behavior occasionally or with prompting, 2: the participant regularly performs the described behavior without assistance). The Adaptive Behavior Composite (ABC) score summarizes performance across all three domains, and domain scores summarized performance across each domain’s three subdomains (see Table 2 for complete list) with lower scores indicating greater impairment.

### Consensus diagnosis of dementia

Participants’ dementia status was determined through expert consensus conference, consisting of at least three staff members who directly observed the participant, as well as a licensed neuropsychologist and neurologist from the parent longitudinal cohort study at UCI [23]. Consensus diagnosis was based upon data obtained through the core assessment battery including direct assessments of participant’s cognitive abilities, and informant reports of participant’s cognitive and functional abilities, maladaptive behaviors, psychiatric symptoms, and general health status. The direct performance-based measures were the Beery-Buktenica Test of Visual-motor integration [51], Wechsler Intelligence Scale for Children-4th Edition Block Design [52], Category Fluency Test [53], DSMSE [25], Haxby Extended Block Design [54], Modified Mini-Mental Status Examination [55], Selective Reminding Test [56], and Test for Severe Impairment [57]. The indirect informant-based measures were the DLD [24], Reiss Screen for Maladaptive Behavior [58], and the VABS-3 [26]. Participants were classified into one of the following six categories: (a) cognitively stable (CS), meaning there was no significant decline in cognitive functioning, (b) mild cognitive impairment (MCI), indicating the presence of mild cognitive or functional decline that did not meet dementia criteria, (c) possible dementia, indicating that there were signs and symptoms of dementia, but the participant’s cognitive decline over time was not convincing enough to be classified as probable dementia status, (d) probable AD dementia status (denoted as Down Syndrome-associated Alzheimer’s disease, DSAD; indicating that AD dementia signs and symptoms were present along with cognitive decline over time, (e) status uncertain due to complications, indicating that criteria for defining dementia were met but undue or concerning circumstances could be connected to the participant’s cognitive status or decline, and (f) indeterminable, indicating that a preexisting disability made it impossible to determine a decline in cognitive status that is indicative of dementia [59]. Due to small sample sizes in the other diagnostic categories (MCI: *n* = 7, possible dementia: *n* = 4), only participants categorized as CS or DSAD were included in analyses to maximize reliability and sensitivity of the study findings. Classification of dementia status was made blinded to sleep questionnaire data.

### Statistical analysis

Differences in demographic and clinical variables by dementia status were assessed using independent samples t-tests for continuous variables and chi-square tests for categorical variables to identify confounds that needed to be adjusted for in primary analyses (see Table 2). As shown in Tables 1 and 2, the DSAD group was older than the CS group [t(50)=–2.09, *p* = 0.042], and had more women [*χ*^2^(1)=4.48, *p* = 0.034]. The two groups did not differ by BMI [t(50)=–0.36, *p* = 0.725], PII [*χ*^2^(1)=0.66, *p* = 0.417], or total impact of stressful events [IMPACT; t(50)=–0.04, *p* = 0.968]. Since age and severity of PII are known to be associated with greater cognitive problems, we adjusted for age, sex [male, female], and PII [mild, moderate and severe] in all primary statistical ANCOVA models examining dementia status group differences [CS, DSAD] in OSA symptoms, insomnia symptom severity across types (sleep onset, maintenance, and terminal), and insomnia symptom severity total scores. Due to the imbalance between diagnostic groups, with the DSAD group being much smaller, bootstrapping was implemented using 5000 samples and bias corrected and accelerated (BCa) intervals to obtain more accurate estimates of confidence intervals and significance values [60]. Significance was determined using an alpha < 0.05 following False Discovery Rate (FDR) correction for multiple comparisons (6 ANCOVA models) [61]. Secondary ANCOVA models additionally adjusted for EVENTS IMPACT and BMI. While all interactions and main effects were tested in these models, only trending and significant effects, at the uncorrected level, are reported in the associated tables. Finally, to verify consistency of findings while minimizing the risk of overfitting, given sample size differences across groups, tertiary nonparametric Mann— Whitney U tests were implemented to examine dementia status group differences [CS, DSAD] in OSA symptoms (i.e., STOP-Bang scores, ESS scores) and insomnia symptom severity across types (sleep onset, maintenance, terminal), and insomnia symptom severity total scores. This last analysis was to confirm consistency of observed effects when not adjusting for confounding variables. Pearson *χ*^2^ tests were also implemented to explore whether the two dementia status groups differed in OSA risk using established cut-offs for STOP-Bang (i.e.,≥3 [38]) and ESS (i.e., >10 [41]). Differences in insomnia symptom severity by PII was also examined using unpaired t-tests to determine if insomnia symptom severity was associated with cognitive impairment unrelated to dementia status. To determine if demographics or sleep disorder symptoms differed by the type of residence, we performed additional non-parametric control analyses due to the imbalance of sample sizes between residence types (i.e., Pearson’s *χ*^2^ test for categorical variables and independent-samples Kruskal-Wallis test for continuous variables).

Partial correlation analyses were next implemented to examine associations between OSA symptoms and insomnia symptom severity with cognitive (DSMSE scores, DLD SCS) and functional activities of daily living (DLD SOS, VABS-3 scores), while adjusting for age, sex, PII, and dementia status (Model 1). Secondary partial correlation analyses additionally adjusted for EVENTS IMPACT and BMI (Model 2). Pearson’s correlation coefficients were also computed to examine consistency of effects when not adjusting for confounding covariates (Model 3). Significance for correlation analyses between sleep and cognitive measures (i.e., DLD SCS and DSMSE scores; 12 models) and sleep and activities of daily living measures (i.e., VABS and DLD SOS scores; 66 models) were determined using an alpha < 0.05 following FDR correction for multiple comparisons [61]. All above-described analyses were completed using SPSS version 26.0 (IBM SPSS Statistics, Inc., Chicago, IL) and R version 3.6.0 [62].

## RESULTS

### OSA-related measures by dementia status

**Fig. 1:**
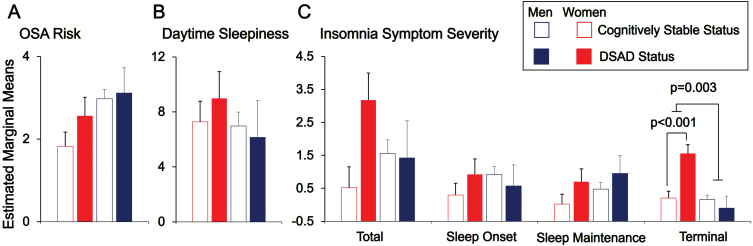
Obstructive sleep apnea (OSA) risk (STOP-Bang scores; A), excessive daytime sleepiness (Epworth Sleepiness Scale scores; B), and Insomnia symptom severity (C) by dementia status and sex in adults with Down syndrome. Bar plots of estimated marginal means in adult men (blue) and women (red) with Down syndrome-associated Alzheimer’s disease (DSAD; solid color) or who were cognitively stable (CS; outlined color) from ANCOVA models with sex [male, female], premorbid intellectual impairment [mild, moderate and severe], and dementia status [CS, DSAD] as between-subjects factors and age as a between-subjects covariates predicting insomnia symptom severity. Following FDR correction across sleep scales (6 comparisons), a significant sex×dementia status interaction term was detected for terminal insomnia symptom severity (*p* = 0.002, FDR *p* = 0.018). Least significant difference (LSD) post hoc testing revealed a significant difference in terminal insomnia symptom severity by dementia status for women (*p* < 0.001) but not for men (*p* = 0.508). Though the sex×dementia status interaction term was not significant for insomnia symptom severity total scores (*p* = 0.080, FDR *p* = 0.240), LSD post hoc testing revealed a significant difference in insomnia symptom severity total scores by dementia status for women (*p* = 0.015) but not for men (*p* = 0.916).

OSA risk (STOP-Bang scores) did not differ significantly by dementia status in adults with DS. An ANCOVA model adjusting for age, sex, and PII was not significant (*F* = 1.6, *p* = 0.320 FDR corrected) and there were no significant predictors of STOP-Bang scores in this model (Table 3, Fig. 1A). Both an ANCOVA model additionally adjusting for EVENTS IMPACT and BMI and an unadjusted non-parametric model (Mann-Whitney U test) also showed no significant difference in OSA risk by dementia status (Supplementary Tables 3 and 4). Further, when analyzed using standardized cut-off scores (≥3 versus <3), there was also no difference by dementia status (*χ*^2^ = 0.66, *p* = 0.417).

**Table 3:** ANCOVA model variables predicting sleep disorder symptoms

Outcome &significant predictors	Model fit (F, p)	Predictor (F, p)	Predictor (FDR p)
**Outcome: STOP-Bang**	1.6, 0.167		0.320
**Predictors:**			
Sex		4.0, 0.052	0.159
**Outcome: ESS**	1.9, 0.082		0.246
**Predictors:**			
Dementia status×Sex×PII		4.3, 0.043	0.240
**Outcome: Insomnia (total)**	1.3, 0.247		0.320
**Predictors:**			
PII		2.9, 0.098	0.202
Dementia status×Sex		3.2, 0.080	0.240
**Outcome: Insomnia (sleep onset)**	0.5, 0.878		0.878
**Predictors:**			
None			
**Outcome: Insomnia (maintenance)**	1.3, 0.267		0.320
**Predictors:**			
Sex×PII		3.6, 0.064	0.285
**Outcome: Insomnia (terminal)**	4.7, 0.0003		0.002
**Predictors:**			
Dementia status		4.1, 0.049	0.234
Sex		11.2, 0.002	0.012
PII		3.7, 0.059	0.202
Dementia status×Sex		10.1, 0.003	0.018
Dementia status×PII		4.5, 0.040	0.240
Sex×PII		2.9, 0.095	0.285
Dementia status×Sex×PII		3.2, 0.080	0.240

Model factors and covariates include age, sex, premorbid intellectual impairment (PII), and dementia status.

Excessive daytime sleepiness (ESS scores) also did not differ significantly by dementia status. An ANCOVA model adjusting for age, sex, and PII was not significant (*F* = 1.9, *p* = 0.246 FDR corrected) and there were no significant predictors of excessive daytime sleepiness in the model (Table 3, Fig. 1B). Both an ANCOVA model additionally adjusting for EVENTS IMPACT and BMI and an unadjusted non-parametric model (Mann-Whitney U test) also did not show a significant difference in excessive daytimes sleepiness by dementia status (Supplementary Tables 3 and 4). Excessive daytime sleepiness also did not differ by dementia status when implementing an established ESS score cut-off (>10; *χ*^2^ = 0.05, *p* = 0.826). Collectively, dementia status in adults with DS was not robustly associated with increased OSA risk nor excessive daytime sleepiness in the current sample.

### Insomnia symptom severity by dementia status

We next examined insomnia symptoms using responses from the CSHQ-A items focused on perceived difficulties in falling asleep (sleep onset), staying asleep (sleep maintenance) and early morning awakening (terminal) between the 2 diagnostic groups (CS, DSAD). ANCOVA models adjusting for age, sex, and PII significantly predicted terminal insomnia symptom severity (*F* = 4.7, *p* = 0.002 FDR corrected) but not sleep onset insomnia symptom severity (*F* = 0.5, *p* = 0.878 FDR corrected),sleep maintenance insomnia symptom severity (*F* = 1.3, *p* = 0.320 FDR corrected), or insomnia symptom severity total scores (*F* = 1.3, *p* = 0.320 FDR corrected; Table 3). In the ANCOVA model predicting terminal insomnia symptom severity, a main effect of sex (*F* = 11.2, *p* = 0.012 FDR corrected) and an interaction effect of sex×dementia status (*F* = 10.1, *p* = 0.018 FDR corrected, Fig. 1 C) were detected. Post hoc testing revealed that dementia status, specifically in adult women with DS, was associated with increased terminal insomnia symptom severity. No other main effects or interaction effects were significant in the model. Findings from both ANCOVA models additionally adjusting for EVENTS IMPACT and BMI and unadjusted non-parametric models (Mann-Whitney U tests) were largely consistent (Supplementary Tables 3 and 4). Collectively, these findings indicate that more frequent insomnia symptoms, particularly terminal insomnia symptoms, are associated with dementia status in adult women with DS, suggesting either AD progression leads to worsening insomnia or insomnia accelerates AD pathophysiology in women with DS.

It is possible that the significant differences in insomnia symptoms between diagnostic groups may not be specific to cognitive impairment resulting from dementia and may exist prior to dementia onset. To address this issue, we examined differences in insomnia symptom severity by degree of premorbid intellectual impairment. No significant effects of premorbid intellectual impairment were detected on insomnia symptom severity total score (*t* = –0.99, *p* = 0.328), sleep onset insomnia symptom severity scores (*t* = –0.18, *p* = 0.857), sleep maintenance insomnia severity scores (*t* = –1.33, *p* = 0.189), or terminal insomnia symptom severity scores (*t* = –0.67, *p* = 0.509), indicating that more frequent insomnia symptoms may be uniquely associated with AD dementia status rather than any form of premorbid cognitive impairment.

To determine if demographics or sleep disorder symptoms differed by the type of residence (i.e., family home, group home, independent), we performed supplementary non–parametric analyses due to the imbalance of sample sizes across residency groups (i.e., Pearson’s *χ*^2^ test for categorical variables and independent-samples Kruskal-Wallis test for continuous variables). With respect to demographics, there were no significant differences by residence type in age (*H* = 2.17, *p* = 0.337), sex (*χ*^2^ = 1.25, *p* = 0.535), dementia status (*χ*^2^ = 3.91, *p* = 0.142), or premorbid intellectual impairment (*χ*^2^ = 3.41, *p* = 0.182). Relevant for secondary models incorporating total impact of stressful events and BMI, residence type was not associated with differences in EVENTS IMPACT (*H* = 0.50, *p* = 0.777) or BMI (*H* = 0.81, *p* = 0.668). In terms of sleep disorder symptoms, there were no significant differences by residence type in STOP-Bang scores (*H* = 2.36, *p* = 0.308), ESS scores (*H* = 0.09, *p* = 0.956), or sleep onset insomnia symptom severity (*H* = 1.56, *p* = 0.459), sleep maintenance insomnia symptom severity (*H* = 1.41, *p* = 0.493), terminal insomnia symptom severity (*H* = 2.73, *p* = 0.255), or insomnia symptom severity total scores (*H* = 0.45, *p* = 0.798). These findings support the interpretation that neither demographic nor sleep disorder symptom variables differed by residence type in this cohort.

### OSA and insomnia symptoms and cognitive impairments

Since there was a dementia status by sex interaction associated with sleep disorder symptoms, we sought to examine which domains of functioning were specifically associated with sleep disorder symptom severity. We first examined associations between current cognitive function and insomnia and OSA symptoms through partial correlation analyses adjusting for age, sex, PII, and dementia status (Table 4). No significant associations were detected between insomnia and OSA measures and cognitive ability (i.e., DSMSE scores) or cognitive impairment (i.e., DLD SCS) following FDR correction. Findings from both partial correlation analyses additionally adjusting for EVENTS IMPACT and BMI as well as unadjusted Pearson’s correlations were largely consistent (Supplementary Tables 5 and 6). Collectively, these findings indicate that OSA risk and insomnia symptoms were not robustly associated with current cognitive impairments in adults with DS while controlling for age, sex, PII, and dementia status.

**Table 4:** Partial correlations

Predictor, Outcome	r	*p*	FDR *p*
**STOP-Bang scores**			
DLD SCS	–0.41	0.005	0.060
**Insomnia scores (total)**			
VABS: Personal	–0.39	0.007	0.057
VABS: Domestic	–0.38	0.009	0.057
VABS: Interpersonal	–0.44	0.002	0.029
VABS: Play &Leisure	–0.49	0.0005	0.029
VABS: Coping	–0.43	0.003	0.029
DLD SOS	0.43	0.003	0.029
**Insomnia scores (sleep onset)**			
VABS: Composite	–0.38	0.008	0.057
VABS: Domestic	–0.35	0.015	0.071
VABS: Play & leisure	–0.36	0.013	0.071
**Insomnia scores (maintenance)**			
VABS: Interpersonal	–0.38	0.009	0.057
VABS: Play & leisure	–0.46	0.001	0.029
VABS: Coping	–0.36	0.014	0.071
**Insomnia scores (terminal)**			
VABS: Personal	–0.44	0.0018	0.029
DLD SOS	0.37	0.010	0.057

Models adjusting for age, sex, premorbid intellectual impairment (PII), and dementia status. Correlations significant or trending after FDR correction are shown.

### OSA and insomnia symptoms and functional activities of daily living

**Fig. 2:**
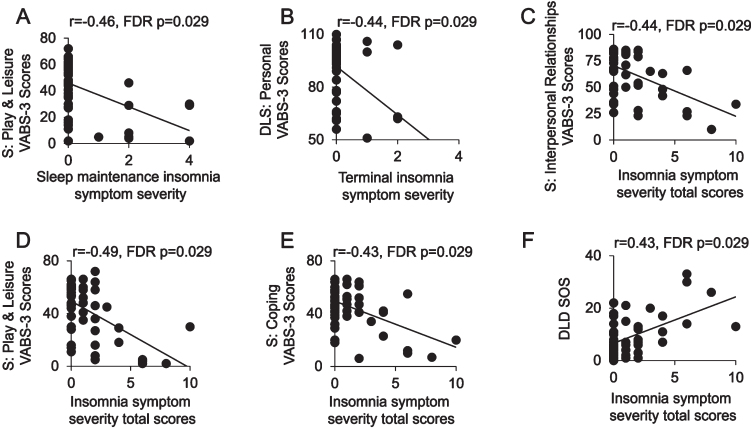
Insomnia symptom severity by adaptive behavioral function and socialization impairments in adults with Down Syndrome. Scatter plots are shown of associations between sleep maintenance (A), terminal insomnia symptom severity (B) and insomnia symptom severity total scores (C–E) and subdomains of the Vineland Adaptive Behavior Scales. 3^rd^ Edition (VABS-3): Play and Leisure (A, D), Personal (B), Interpersonal Relationships (C), and Coping skills (E). An additional scatter plot (F) is shown of insomnia symptom severity total scores predicting Dementia Questionnaire for People with Learning Disabilities (DLD) Sum of Social scores (SOS). FDR, false discovery rate multiple comparisons correction method; DLS, Daily Living Skills; S, Socialization.

We lastly examined associations between sleep disorder symptoms and functional impairments, which were necessary for a consensus diagnosis of dementia. Primary partial correlation analyses between sleep disorder symptoms and functional measures of activities, adjusting for age, sex, PII, and diagnostic group (Table 4) were performed. There were no significant correlations between OSA risk (all r^2^ < 0.093, *p* > 0.14 FDR corrected) or excessive daytime sleepiness (all *r*^2^ < 0.05, *p* > 0.3 FDR corrected) and functional activities (i.e., VABS-3 scores and DLD SOS scores) following FDR correction. In contrast, insomnia symptom severity was significantly and specifically associated with impairment in activities of daily living and social functioning (Fig. 2). Specifically, higher insomnia symptom severity total scores were negatively correlated with multiple Vineland subdomain scores (VABS-3 Interpersonal *r* = –0.44, *p* = 0.029 FDR corrected; VABS-3 Play and Leisure *r* = –0.49, *p* = 0.029 FDR corrected; VABS-3 Coping Skills *r* = –0.43, *p* = 0.029 FDR corrected) and positively associated with impairments in social functioning (DLD SOS *r* = 0.43, *p* = 0.029 FDR corrected). Sleep maintenance insomnia symptom severity was negatively correlated with a subscale of socialization (VABS-3 Play and Leisure *r* = –0.46, *p* = 0.029 FDR corrected). Lastly, terminal insomnia symptom severity was negatively correlated with a measure of daily living skills (VABS-3 Personal *r* = –0.44, *p* = 0.029 FDR corrected). Findings from both partial correlation analyses additionally adjusting for EVENTS IMPACT and BMI as well as unadjusted Pearson’s correlations were largely consistent (Supplementary Tables 5 and 6). Together, these findings demonstrate that insomnia symptoms were more closely associated with a lower level of daily living skills than impaired cognition.

## DISCUSSION

Our study in older adults with DS found that terminal insomnia symptoms, defined as waking up earlier than necessary or desired in the morning and having difficulty falling back asleep, were more severe in women with AD dementia than in women who were cognitively stable. Insomnia symptoms were more robustly associated with functional impairments in activities of daily living than cognitive impairments, even after adjusting for age, sex, premorbid intellectual impairment, and dementia status in partial correlation analyses. Further, these associations were not observed with measures of OSA risk or excessive daytime sleepiness, suggesting that the deterioration in the ability to perform everyday activities of living may be more closely related to symptoms of insomnia than sleep-disordered breathing in DS. Collectively, these data identify insomnia as a factor relevant for functional deterioration and AD dementia status in adults with DS, especially for women, underscoring the potential importance of insomnia screening and/or treatment in this population.

Most studies to date in adults with DS have focused on OSA prevalence [18, 20, 63–70], leading to a general paucity of data regarding insomnia prevalence in this population. Regardless of sleep disorder, little is known about the effect of AD progression on the prevalence of sleep problems or the effect of sleep disorders on the progression of AD in adults with DS. In neurotypical older adult populations, insomnia symptoms, presence of OSA, and excessive daytime sleepiness are associated with increased burden of AD pathologies and/or risk for dementia [13–15, 71, 72]. Accumulated evidence in neurotypical populations indicates that while OSA increases risk for all causes of dementia, insomnia may specifically increase risk for AD [71], supporting the possibility that OSA and insomnia may be associated with AD through distinct mechanisms. Our findings support this hypothesis, with insomnia but not OSA symptoms being associated with functional impairments linked with AD progression in other studies in adults with DS [73–76]. This may be due to insomnia symptoms acting as an early sign of cognitive deterioration, which ultimately impacts the ability to perform activities of daily living. While speculative, another possibility is that insomnia pathophysiology is associated more specifically with AD pathology [77], while OSA may be associated with multiple pathophysiological factors more common across dementias, including vascular pathologies [13, 77–79]. This is supported by a recent report that shows that the presence of OSA in adults with DS increased the magnitude of the difference in white matter hyperintensity (WMH) volumes across AD diagnostic groups [80]. This possibility is also supported by a recent study showing an association between actigraphy-measured duration of nighttime awakenings, a common clinical feature of insomnia [81, 82], and AD pathology in adults with Down syndrome without dementia [83]. However, other studies show clear links between OSA and AD pathologies [13], including in adults with DS [80], indicating that multiple forms of sleep disturbance may impact AD pathophysiology in adults with DS. Possible links between insomnia, OSA, and AD pathophysiology in adults with DS must be tested in future studies and with larger samples to address these possibilities, especially given the mechanistic role of APP triplication on amyloid-β overproduction and AD in adults with DS [84, 85].

The current study identified an effect of sex on the relationship between AD dementia and terminal insomnia symptoms. While sex differences in insomnia symptoms have not been examined in adults with DS, this finding is not surprising given the established literature in neurotypical populations. Insomnia symptoms increase with age, and this increase is larger in women than in men [86–88]. Further, women are more likely than men to exhibit multiple different kinds of insomnia symptoms in later life [89]. In the context of dementia, insomnia symptoms increase the risk for developing AD dementia [71] and are highly prevalent in dementia [7]. In children and adolescents with DS, insomnia symptoms are associated with behavioral and social dysfunction [90]. This might be particularly concerning for older women with DS, since, at least in neurotypical populations, relationships between insomnia symptoms and psychological measures of distress are more sustained into older age in women than men [91]. Collectively, these findings support the hypothesis that insomnia symptoms may be an important risk factor for AD dementia in adult women with DS.

In contrast to our findings with insomnia symptoms, our study did not find symptoms of OSA risk to be significantly increased in participants with probable AD dementia, consistent with a recent report using personal health history and medical records to determine the presence/absence of OSA [80]. One likely possibility is that STOP-Bang scores, while sufficiently accurate and sensitive to the presence or absence of OSA, are not sensitive to the severity of OSA disease features that may be more directly tied to the pathophysiology of AD, such as the severity of hypoxemia or sleep fragmentation. In other words, it may not be whether you have OSA or not that matters for AD but rather the severity of the pathophysiological features of OSA. Future studies incorporating objective measures of OSA disease severity using polysomnography are needed to further evaluate the role of OSA in dementia pathophysiology in DS. It is also possible that there is a ceiling effect, as it has been estimated that between 65–100% of adults with DS have OSA [21]. Yet another possibility stems from the low prevalence of hypertension in DS [92, 93], which has been shown to be a critical mechanism linking OSA to cognitive decline and AD pathophysiology [94]. This could indicate that while OSA is prevalent in DS, its effects on dementia pathophysiology and cognitive function may be blunted relative to normative populations. Follow-up studies are necessary to examine this possibility more closely. Regardless of their potential influence on dementia risk, OSA symptoms and excessive daytime sleepiness were not associated with deterioration in adaptive behavior skills, highlighting the possibility of an insomnia-specific relationship with the progression of AD in female adults with DS.

The current study identified associations between insomnia symptoms and social and behavioral dysfunction and dementia status, particularly in older women with DS, but did not find associations between these factors and excessive daytime sleepiness. At first glance, this may appear to be paradoxical, as one may expect that caregivers reporting insomnia symptoms would also report symptoms of excessive daytime sleepiness. However, insomnia is often associated with inappropriate hyperarousal that persists throughout the 24-hour day, limiting symptoms of daytime sleepiness [95, 96]. For example, studies in chronic insomnia patients consistently demonstrate increased daytime sleep latency on the Multiple Sleep Latency Test— a standardized in-lab protocol to assess daytime sleepiness objectively— even in the presence of reduced objectively measured nighttime sleep duration [95, 97]. While daytime fatigue is one of the commonly reported functioning impairments associated with nighttime sleep difficulties in insomnia, most patients with insomnia disorder report having difficulties sleeping during the day even when being provided with sufficient sleep opportunities [98], indicating that symptoms of fatigue and sleepiness are distinct constructs. For this reason, we think our findings are consistent with the established literature in showing behavioral and social functioning is associated with insomnia symptoms but not with daytime sleepiness.

Despite the strength of the findings, this study had limitations. First, sleep data were based upon subjective reports from caregivers, limiting sensitivity to detect sleep disturbance unless it is associated with behavioral disruptions observed by caregivers. Of note, the current cohort included individuals with DS with various forms of residency, and the type of residence may have impacted the accurate reporting of sleep disorder symptoms. While residence type did not have a statistical effect on demographic, sleep disorder symptom, or dementia status variables, we cannot rule out the possibility that sleep disorder symptoms are worse in participants living in group homes or that informants for participants living independently or in the family home are underreporting sleep disorder symptoms because they are not with the participant all night or awake themselves. It is also possible that living in a group home and with a roommate or with multiple housemates can lead to more sleep disturbances that are due to environmental causes. Further, while subjective reports are a well-validated approach for measuring insomnia symptomatology, using objective OSA measures may improve sensitivity to detect relationships with dementia risk factors, though STOP-Bang scores have been validated against clinical polysomnography [38, 39]. Of concern is whether informants report more reliable data on certain forms of insomnia symptoms than other forms or compared to symptoms of OSA in adults with DS, further underscoring the importance of objective sleep measures to assess true relationships between sleep disturbance and dementia in adults with DS. However, we do think the OSA-related measures chosen for the current study are less vulnerable to this issue. For example, excessive daytime sleepiness can be reliably reported, as these symptoms are regarding the likelihood of dozing during the day when the informants are most likely to interact with and observe the participants. Moreover, many of the metrics comprising the STOP-Bang scores, including daytime tiredness, high blood pressure, BMI > 35 kg/m^2^, age > 50 years, neck size > 16 inches (which was measured objectively in the current study), and male sex are possible to collect with high degree of accuracy from informants. It nevertheless remains true that objective measures of OSA expression will offer important insights into the biological associations between OSA and dementia in adults with DS, and this should be examined in future studies. Measuring biological markers of AD pathophysiology would enhance AD diagnostic specificity and mechanistic understanding of the role of sleep disturbances in AD specifically in DS. Lastly and building on our current findings, future studies focused on sex effects on sleep-associated links with dementia-related dysfunction would be important to better understand potential sex differences in AD risk in adults with DS.

In conclusion, terminal insomnia symptoms are more severe in adult women with DS that have dementia relative to those that do not. Moreover, regardless of sex, insomnia symptoms were associated with greater functional, but not cognitive, impairments, whereas OSA symptoms and excessive daytime sleepiness were not significantly related to either. Insomnia symptoms may thus be an important underrecognized form of sleep disturbance in adults, and maybe particularly in adult women, with DS that is relevant for assessing dementia progression as either an early symptom of cognitive or psychiatric dysregulation or a factor contributing to disease mechanisms. While the findings of the current study are based on subjective measures of sleep problems, they identify important candidate targets for future studies using objective sleep measures. Furthermore, since sleep disorders, including insomnia, OSA, and circadian rhythm disturbances, are readily treatable, it will be important to study whether sleep disorder treatment impacts functional status, AD pathophysiology, and ultimately, AD risk in adults with DS.

## AUTHOR CONTRIBUTIONS

Shivum Desai (Data curation; Investigation; Writing – original draft); Ivy Y. Chen (Conceptualization; Data curation; Formal analysis; Methodology; Resources; Visualization; Writing – review & editing); Christy Hom (Conceptualization; Resources; Supervision; Writing – review & editing); Eric Doran (Data curation; Investigation; Methodology; Writing – review & editing); Dana D. Nguyen (Data curation; Investigation; Writing – review & editing); Ruth M. Benca (Conceptualization; Supervision; Writing – review & editing); Ira T. Lott (Conceptualization; Funding acquisition; Project administration; Resources; Supervision; Writing – review & editing); Bryce A. Mander (Conceptualization; Formal analysis; Funding acquisition; Methodology; Project administration; Resources; Supervision; Visualization; Writing – original draft; Writing – review & editing).

## Supplementary Material

Supplementary Material

## Data Availability

The data that support the findings of this study are available from the corresponding author upon request following review and approval by co-author Dr. Lott. The data are not publicly available due to privacy or ethical restrictions. ABC-DS data are available to qualified researchers by request at https://www.nia.nih.gov/research/abc-ds.
