# Supplementary material for: Insomnia Symptoms Are Associated with Measures of Functional Deterioration and Dementia Status in Adults with Down Syndrome at High Risk for Alzheimer’s Disease
Source: J Alzheimers Dis. 2024 Jul 16;100(2):613–29. doi: 10.3233/JAD-220750 (PMC11307085; doi:10.3233/JAD-220750)
Supplement: Supplementary Material [file jad-100-jad220750-s001.pdf]

Supplementary Material

Insomnia Symptoms Are Associated with Measures of Functional Deterioration and Dementia Status in Adults with Down Syndrome at High Risk for Alzheimer's Disease

Supplementary Table 1. ABC-DS Cycle 1 visit versus Cohort demographic characteristics

	Study Cohort (n=52)	ABC-DS (n=304)		
	n (%)	n (%)	t, c^2	p
Consensus Diagnosis				
No MCI or dementia (n; %)	40 (76.9)	259 (85.2)	2.25	0.133
Dementia (n; %)	12 (23.1)	45 (14.8)		
Age (y)	52.2±6.4	43.9±9.8	5.88	<0.001
Sex				
Male (n; %)	31 (59.6)	158 (52.0)	1.03	0.309
Female (n; %)	21 (40.4)	146 (48.0)		
PII				
Mild (n; %)	25 (48.1)	163 (53.8)	2.23	0.328
Moderate (n; %)	24 (46.2)	110 (36.3)		
Severe (n; %)	3 (5.8)	30 (9.9)		
Residence type				
With family/caregiver (n; %)	26 (50)	155 (51.7)	0.17	0.919
Group home (n; %)	20 (38.5)	107 (35.7)		
Independent (n; %)	6 (11.5)	38 (12.7)		
Day program				
Yes (n; %)	31 (59.6)	168 (56.8)	0.15	0.703
No (n; %)	21 (40.4)	128 (43.2)		
Community paid job				
Yes (n; %)	10 (19.2)	105 (35.5)	5.28	0.022
No (n; %)	42 (80.8)	191 (64.5)		

ABC-DS. Alzheimer's Biomarkers Consortium — Down Syndrome Cycle 1 Visit; PII, premorbid intellectual impairment

Only participants designated as having "No MCI or dementia" or as having "Dementia" were selected from the ABC-DS consortium for comparison purposes.

Supplementary Table 2. ABC-DS Cycle 2 versus Cohort demographic characteristics

	Study Cohort (n=52)	ABC-DS (n=246)		
	n (%)	n (%)	t, c^2	p
Consensus Diagnosis				
No MCI or dementia (n; %)	40 (76.9)	188 (76.4)	0.01	0.939
Dementia (n; %)	12 (23.1)	58 (23.6)		
Age (y)	52.2±6.4	45.9±9.6	4.50	<0.001
Sex				
Male (n; %)	31 (59.6)	123 (50.0)	1.58	0.209
Female (n; %)	21 (40.4)	123 (50.0)		
PII				
Mild (n; %)	25 (48.1)	136 (55.3)	3.99	0.136
Moderate (n; %)	24 (46.2)	81 (32.9)		
Severe (n; %)	3 (5.8)	29 (11.8)		
Residence				
With family/caregiver (n; %)	26 (50)	120 (49.0)	0.01	0.995
Agency/group home (n; %)	20 (38.5)	96 (39.2)		
Independent (n; %)	6 (11.5)	28 (11.4)		
Day program				
Yes (n; %)	31 (59.6)	141 (57.6)	0.07	0.786
No (n; %)	21 (40.4)	104 (42.4)		
Community paid job				
Yes (n; %)	10 (19.2)	84 (34.3)	4.50	0.034
No (n; %)	42 (80.8)	161 (65.7)		

ABC-DS, Alzheimer's Biomarkers Consortium — Down Syndrome Cycle 2; PPI, premorbid intellectual impairment

Only participants designated as having “No MCI or dementia” or as having “Dementia” were selected from the ABC-DS consortium for comparison purposes.

Supplementary Table 3. Full ANCOVA model variables predicting sleep disorder symptoms

Outcome & significant predictors	Model fit (F, p)	Predictor (F, p)	Predictor (FDR p)
**Outcome: STOP-Bang**	5.0, <0.001		0.0003
**Predictors:**			
BMI		29.3, <0.001	<0.001
Sex		6.0, 0.019	0.057
Age		4.5, 0.041	0.246
**Outcome: ESS**	1.8, 0.084		0.126
**Predictors:**			
Dementia status×Sex×PII		5.4, 0.025	0.075
**Outcome: Insomnia (total)**	2.9, 0.008		0.016
**Predictors:**			
Total impact of stressful events		15.0, <0.001	0.002
Dementia status		3.6, 0.065	0.195
Dementia status×Sex		4.6, 0.039	0.117
**Outcome: Insomnia (sleep onset)**	1.3, 0.257		0.257
**Predictors:**			
Total impact of stressful events		8.8, 0.005	0.015
**Outcome: Insomnia (maintenance)**	1.6, 0.143		0.172
**Predictors:**			
Total impact of stressful events		4.6, 0.038	0.057
**Outcome: Insomnia (terminal)**	5.0, <0.001		<0.001
**Predictors:**			
Total impact of stressful events		7.3, 0.010	0.020
Dementia status		5.2, 0.018	0.108
Sex		12.9, <0.001	0.005
Dementia status×Sex		11.5, <0.001	0.012
Sex×PII		7.0, 0.011	0.066
Dementia status×Sex×PII		6.1, 0.018	0.075

Model factors and covariates include age, sex, premorbid intellectual impairment (PII), dementia status, total impact of stressful events (IMPACT), and body mass index (BMI)

Supplementary Table 4. Mann-Whitney model variables predicting sleep disorder symptoms

Outcome	Test statistic	p
STOP-Bang scores	0.83	0.406
ESS scores	0.44	0.663
Insomnia scores (total)	1.16	0.248
Insomnia scores (sleep onset)	-0.17	0.867
Insomnia scores (maintenance)	0.96	0.339
Insomnia scores (terminal)	2.08	0.038

Supplementary Table 5. Partial correlations

Predictor, Outcome	r	p	FDR p
**Insomnia scores (total)**			
VABS: Personal	-0.41	0.006	0.049
VABS: Domestic	-0.44	0.003	0.030
VABS: Interpersonal	-0.45	0.002	0.030
VABS: Play & Leisure	-0.48	0.0007	0.025
VABS: Coping	-0.40	0.007	0.0498
DLD SOS	0.52	0.0003	0.019
**Insomnia scores (sleep onset)**			
VABS: Composite	-0.36	0.016	0.088
VABS: Domestic	-0.38	0.010	0.064
**Insomnia scores (maintenance)**			
VABS: Interpersonal	-0.36	0.016	0.088
VABS: Play & Leisure	-0.44	0.003	0.030
**Insomnia scores (terminal)**			
VABS: Personal	-0.44	0.002	0.030
DLD SOS	0.40	0.006	0.049

Models adjusting for age, sex, premorbid intellectual impairment (PII), dementia status, total impact of stressful events (IMPACT), and body mass index (BMI)
Correlations significant or trending after FDR correction are shown.

Supplementary Table 6. Pearson's correlations

Predictor, Outcome	r	p	FDR p
**Insomnia scores (total)**			
VABS: Composite	-0.31	0.026	0.065
VABS: Receptive language	-0.41	0.002	0.011
VABS: Written language	-0.35	0.011	0.034
VABS: Personal	-0.51	0.0001	0.001
VABS: Domestic	-0.45	0.001	0.004
VABS: Community	-0.33	0.017	0.045
VABS: Interpersonal	-0.51	0.0001	0.001
VABS: Play & Leisure	-0.55	0.00002	0.0005
VABS: Coping	-0.50	0.0002	0.001
DLD SCS	0.36	0.009	0.052
DLD SOS	0.51	0.0001	0.001
**Insomnia scores (sleep onset)**			
VABS: Play & Leisure	-0.35	0.010	0.032
**Insomnia scores (maintenance)**			
VABS: Personal	-0.36	0.009	0.032
VABS: Domestic	-0.34	0.013	0.037
VABS: Interpersonal	-0.39	0.004	0.021
VABS: Play & Leisure	-0.47	0.0005	0.003
VABS: Coping	-0.39	0.005	0.021
DLD SOS	0.36	0.008	0.030
**Insomnia scores (terminal)**			
VABS: Receptive language	-0.56	0.00002	0.0005
VABS: Expressive language	-0.38	0.005	0.021
VABS: Written language	-0.34	0.014	0.039
VABS: Personal	-0.62	0.000001	0.00008
VABS: Domestic	-0.37	0.007	0.026
VABS: Community	-0.29	0.039	0.094
VABS: Interpersonal	-0.47	0.0004	0.003
VABS: Play & Leisure	-0.35	0.010	0.032
VABS: Coping	-0.50	0.0002	0.001
DLD SCS	0.41	0.002	0.029
DLD SOS	0.53	0.00005	0.0008

Correlations significant or trending after FDR correction are shown.
